# Roadmap for Linking Registry Data with Health Services Data to Support Evidence-Informed Decision-Making.

**DOI:** 10.23889/ijpds.v9i5.2755

**Published:** 2024-09-18

**Authors:** Magda Nunes de Melo, Anne Hayes, Charles Victor, Laurie Lambert, Trish Caetano, Nicole Mittmann

**Affiliations:** 1 ICES; 2 HDRN Canada; 3 CADTH

## Objective and Approach

As part of a learning period to optimize the use of RWE for decision-making for drugs for rare diseases, the [organization name removed to allow for blind review] conducted an environmental scan to map real-world data in patient registries. Over 400 patient registries were identified, signaling the potential wealth of untapped information to support decision-making by linking registry data with health services data. To better understand the challenges faced by registry holders hoping to link registry data with health services data sources available in Canada, a series of interviews were conducted with several Canadian rare disease registries. In addition, a literature review was completed, and Canadian experts in epidemiology, privacy, record linkage, registry science, and health services research were consulted to inform the development of a roadmap to meet various stakeholder needs.

## Results

The resulting roadmap consists of 8 specific steps covering topics related to registry purpose, informed consent, ethical approval, participant privacy, governance, data linkability, participant identifiability and jurisdictional requirements.

## Conclusion

The roadmap is currently undergoing pilot testing by a pan-Canadian rare disease registry. A final [organization name] report and an accompanying roadmap in a checklist format to facilitate implementation will be finalized, disseminated across key stakeholders, and made publicly available.

## Implications

While developed for registries, the roadmap applies to the linkage of clinical trial or cohort study data, or other systematically gathered patient-level data to health services data.

